# Prevalence of cardiovascular risk factors and their association with renal impairment in elderly patients with type 2 diabetes mellitus in a Nigerian tertiary hospital: a cross-sectional study

**DOI:** 10.11604/pamj.2022.42.233.35265

**Published:** 2022-07-26

**Authors:** Oladimeji Adedeji Junaid, Olubukola Ayoola Ojo, Oluseyi Ademola Adejumo, Folorunsho Mansally Junaid, Sunday Samson Owolade, Olalekan Ezekiel Ojo, Babatope Ayodeji Kolawole, Temidayo Rosemary Ikem

**Affiliations:** 1 Department of Internal Medicine, University of Medical Sciences, Ondo State, Nigeria,; 2 Department of Internal Medicine, Federal Medical Centre, Owo, Ondo State, Nigeria,; 3 The Royal Wolverhampton NHS Trust, Wolverhampton, United Kingdom,; 4 Department of Internal Medicine, Obafemi Awolowo University, Ile-Ife, Osun State, Nigeria

**Keywords:** Elderly, type 2 diabetes mellitus, cardiovascular risk factor, renal impairment

## Abstract

**Introduction:**

the population of elderly with Type 2 Diabetes Mellitus (T2DM) has been on the increase. The burden of cardiovascular disease and renal impairment may also increase due to the relationship between cardiovascular risk factors and ageing in those with T2DM. The prevalence of cardiovascular risk factors and their association with renal impairment in elderly with T2DM were determined.

**Methods:**

this is a cross-sectional study that involved 96 elderly patients with T2DM and 96 elderly individuals without DM as control. The prevalence of cardiovascular risk factors was determined among the study participants. Binary logistic regression was used to determine the significant cardiovascular factors associated with renal impairment among the elderly with T2DM. P-value <0.05 was taken as significant.

**Results:**

the mean age of the elderly with T2DM and control group were 66.73±5.18 years and 66.78±5.25years, respectively. The male: female ratio was 1: 1 for both groups. The prevalence of the cardiovascular risk factors in the elderly with T2DM and control were; hypertension (72.9%vs39.6%; p ≤0.001), high glycated haemoglobin (77.1% vs 0%; p ≤0.001), generalized obesity (34.4%vs1.0%; p ≤0.001), central obesity (50.0%vs11.5%; p ≤0.001), dyslipidemia (97.9%vs89.6%; p=0.016), albuminuria (69.8% vs 11.2%; p ≤0.001), anaemia (53.1%vs18.8%; p ≤0.001). Renal impairment was present in 44.8% of the elderly T2DM. On multivariate analysis, the cardiovascular risk factors significantly associated with renal impairment in elderly with T2DM were high glycated haemoglobin (aOR: 6.21, 95% CI: 1.61-24.04; p=0.008), albuminuria (aOR: 4.77, 95% CI: 1.59-14.31; p=0.005) and obesity (aOR: 2.78, 95%CI 1.04-7.45; p=0.042).

**Conclusion:**

cardiovascular risks factors were highly prevalent and closely associated with renal impairment in elderly with T2DM. Early cardiovascular risk factor modification may reduce both renal and cardiovascular disease burden.

## Introduction

There has been a significant increase in the global population of elderly in the recent years which is partly due to improvement in health care services [1]. In the United States, the elderly population was reported to constitute 16% of the total population in 2019 [2]. In Nigeria, the population of elderly increased by more than 4 folds between 1960 and 2020 [3]. The World Health Organization (WHO) projected that the elderly population will double from 12% to 22% between year 2015 and 2050; and 80% of elderly population will be in the low and middle-income countries [1]. One of the possible implications of this epidemiologic transition is an increase in cardiovascular disease burden. This is because ageing is associated with increased cardiovascular risk [4].

Cardiovascular disease is the leading cause of mortality in the elderly population [5,6]. Hypertension and diabetes mellitus (DM) are common risk factors associated with these cardiovascular diseases and they are highly prevalent in the older adults [6,7]. In a systematic review and meta-analysis of studies on hypertension in older adults in Africa, the pooled prevalence of hypertension was reported to be 57% by Bosu *et al*. [7]. Sinclair *et al*. [8] reported that 20% of elderly have DM while another 20% have undiagnosed DM. DM is associated with increased risk of cardiovascular disease and renal dysfunction especially in elderly [9-11]. The underlying link between these complications is cardiovascular risk factors such as hypertension, albuminuria, dyslipidemia and metabolic syndrome which are highly prevalent in those with DM [12,13]. The interplay of these factors is responsible for the high burden of cardiovascular and renal disease among those with DM [12,14]. According to previous studies, the burden of kidney disease in elderly with DM is high; ranging between 20.8-59.5% depending on the mode of assessment of the kidney function such as albuminuria, reduction in estimated glomerular filtration or a combination of both [10,11,15]. Other factors that may also determine the degree of renal impairment in individuals with DM include age, body mass index, glycemic control, blood pressure control and genetic factors which may vary in these studies [10,11,15,16].

There is limited literature on cardiovascular risk factors in elderly with DM in Africa including Nigeria. Majority of the existing studies on cardiovascular risk factors in DM in Africa were done among young and middle age population, despite being the leading cause of mortality in the elderly. In addition, the practice of geriatric medicine is still evolving in Nigeria. Early cardiovascular risk assessment and modification in the elderly with DM will reduce their cardiovascular and renal morbidity and mortality. It is against this background that this study determined and compared the prevalence of cardiovascular risk factors among elderly patients with type 2 DM (T2DM) and those without DM. It also determined the association between renal impairment and these cardiovascular risk factors.

## Methods

**Study design and setting**: this was a cross-sectional study that was conducted over a 6-month period between December, 2016 and May 2017. The study was carried out at the Federal Medical Centre, Owo which is a tertiary health facility located in Ondo State, Southwest Nigeria and receives referral of patients from within and outside the State.

**Study population**: the study population was made up of elderly T2DM patients presenting at the endocrinology outpatient clinic of the Federal Medical Centre, Owo. Age and sex matched elderly men and women without DM from the general outpatient department of the hospital were recruited as controls. Inclusion criteria included consenting T2DM patients who were 60 years and above diagnosed to have T2DM for a minimum period of 6 months. Inclusion criteria for control included consenting individuals who were 60 years and above without DM or glucose intolerance. Those with chronic illness such as Human Immunodeficiency Virus infection, heart failure, thyroid disorders and chronic obstructive pulmonary disease were excluded from the study. The minimum sample size for this study was calculated using Fisher´s formula; [17] with a reported prevalence of DM in elderly taken as 6% reported in a previous study; [18] and an absolute precision limit of 5%. The minimum sample size after inclusion of 10% attrition was 96. A total of 96 elderly T2DM patients and 96 elderly individuals without DM who fulfilled the inclusion criteria were consecutively recruited for the study.

**Data collection**: an interviewer administered questionnaire was used to obtain the demographic characteristics, medical and social history of the study participants. All study participants were examined. Blood pressure values were taken following standard procedure. Weight and height were measured using a stadiometer with participants wearing light clothing and without shoes. Body Mass Index (BMI) was calculated using the formula weight/height^2^ expressed in unit of kg/m^2^. Waist circumference was measured mid-way between the inferior margin of the last rib and iliac crest using inelastic tape maintained in a horizontal plane at the end of gentle expiration with feet kept 20-30cm apart. Ten mls of fasting blood was taken for packed cell volume, serum creatinine, lipid profile, and glycated hemoglobin. Spot urine was assessed for albumin-creatinine ratio (ACR).

**Definitions**: central obesity was defined as waist circumference (WC) ≥ 88cm for females and ≥ 102cm for males [19]. Generalized obesity was defined as BMI ≥ 30 kg/m^2^ [20]. Dyslipidemia was defined as any or combination of the following: total cholesterol >200 mg/dl; high-density lipoprotein cholesterol (HDL-C) <50 mg/dl in females and <40 mg/dl in males; low-density lipoprotein cholesterol (LDL-C) >130 mg/dl; and triglyceride (TG) >150 mg/dl [21]. Anaemia was defined as packed cell volume (PCV) <36% in females and <39% in males using the WHO criteria [22]. Albuminuria was defined as ACR of >30mg/g [23]. Good glycemic control was defined as glycated hemoglobin < 7.0% [24]. Renal impairment was defined as a glomerular filtration rate (GFR) less than 60mls/mins 1.73m^2^ [25]. Estimated GFR was calculated using modification of diet in renal disease (MDRD) formula that has been previously validated in Nigerians [26]. Elderly was defined as those who were 60 years and above [27].

**Ethical consideration**: informed consent was obtained from all participants and their information were treated with confidentiality. Ethical approval with protocol number: FMC/OW/380/VOL.XLII/185 was obtained from the human research and ethics committee of Federal Medical Centre, Owo, Ondo State for this study.

**Statistical analysis**: data obtained were entered and analyzed using the Statistical Package for Social Sciences (SPSS) version 21.0 software 9 (IBM-SPSS, Armonk, NY: IBM Corporation). Descriptive data were presented as tables and categorical variables of the two groups were expressed as proportions and percentages. Association between categorical variables was analyzed using Chi-square. Fisher´s exact test was used when the number of count was less than 5. Binary logistic regression was used to determine adjusted odds ratio (aOR) and 95% confidence interval (CI) in order to find the significant factors associated with renal impairment in elderly with T2DM. Variables with p-values < 0.05 at the bivariate level were entered into the multivariable analysis. The level of significance for each test was set at p < 0.05.

## Results

The study participants were made up of 96 elderly patients with T2DM and 96 sex and aged matched elderly individuals without DM. Their mean age values were 66.73±5.18 years and 66.78±5.25 years, respectively. Each group was made of 48(50%) males and 48(50%) females. Majority of the study participants were within the age group of 60-69 years. About one-third of the study participants had tertiary education and were retirees (Table 1). The prevalence of the cardiovascular risk factors among elderly with T2DM and the control group were hypertension (72.9%vs39.6%; p ≤0.001), high glycated haemoglobin (77.1%vs0%; p ≤0.001), generalized obesity (34.4%vs1.0%; p ≤0.001), central obesity (50%vs11.5%; p ≤0.001), anaemia (53.1%vs18.8%; p ≤0.001), dyslipidaemia (97.9%vs 89.6%; p=0.016); and albuminuria (69.8%vs11.4%; p ≤0.001). The prevalence of renal impairment in the elderly with T2DM was 44.8% while none of the elderly in the control group had renal impairment (Table 2).

**Table 1:** socio-demographic and clinical characteristics of study participants

Socio-demographic Variables	Elderly with T2DM n (%) N = 96	Elderly without T2DM n (%) N = 96	P-value
**Age in years**			
(Mean±SD)	66.73±5.18	66.78±5.25	0.902
60-69	71(74.0)	70(72.9)	
70-79	22(22.9)	21(21.9)	0.767
>79	3(3.1)	5(5.2)	
**Gender**			
Males	48(50)	48(50)	
Females	48(50)	48(50)	1.000
**Educational status**			
None	29(30.2)	29(30.2)	
Primary	27(28.2)	20(20.8)	0.486
Secondary	10(10.4)	16(16.6)	
Tertiary	30(31.2)	31(32.2)	
**Occupation**			
Retiree	37(38.6)	33(34.4)	
Trading	24(25.0)	19(19.8)	
Farming	20(20.8)	23(24.0)	0.685
Artisans	8(8.3)	13(13.5)	
Civil servants	7(7.3)	8(8.3)	
**Marital status**			
Married	72(75.0)	74(77.0)	
Widow/widower	20(20.8)	20(20.8)	0.707
Separated/divorced	4(4.2)	2(2.2)	

T2DM (type 2 diabetes mellitus)

**Table 2:** comparison between cardiovascular risk factors among study participants

Cardiovascular Risk Factor	Elderly with T2DM n (%) N = 96	Elderly without T2DM n (%) N = 96	P-value
**Hypertension**			
Present	70(72.9)	36(39.6)	<0.001
Absent	26(27.1)	58(60.4)	
**Glycemic Control**			
<7%	22(22.9)	96(100)	<0.001
≥7%	74(77.1)	0(0)	
**Generalized Obesity**			
Present	33(34.4)	1(1.0)	<0.001
Absent	63(65.6)	55(99.0)	
**Central Obesity**			
Present	48(50.0)	11(11.5)	<0.001
Absent	48(50.0)	85(88.5)	
**Dyslipidemia**			
Present	94(97.9)	86(89.6)	0.016
Absent	2(2.1)	10(10.4)	
**Albuminuria**			
Present	67(69.8)	11(11.4)	<0.001
Absent	29(30.2)	85(88.5)	
**Anemia**			
Present	51(53.1)	18(18.8)	<0.001
Absent	45(46.9)	78(81.2)	
**Low GFR**			
Present	43(44.8)	0(0.0)	<0.001
Absent	53(55.2)	98(100.0)	

T2DM (type 2 diabetes mellitus), GFR (glomerular filtration rate)

Cardiovascular risk factors significantly associated with renal impairment in elderly T2DM patients were high glycated haemoglobin (p ≤0.001), generalized obesity (p= 0.021) and albuminuria (p=0.002). On multivariate analysis, the cardiovascular risk factors that were significantly associated with renal impairment in elderly with T2DM patients were high glycated haemoglobin (aOR: 6.21, 95% CI: 1.61-24.04; p=0.008); albuminuria (aOR: 4.77, 95% CI: 1.59-14.31; p=0.005) and generalized obesity (aOR: 2.78,95% CI 1.04-7.45; p=0.042) (Table 3).

**Table 3:** association between renal Impairment and cardiovascular risk factors in elderly with T2DM

Cardiovascular Risk Factor	GFR<60mls/minn(%)	GFR≥60mls/min n(%)	OR(95% CI)	P-value	aOR (95% CI)	P-value
**Hypertension**						
Present	35(81.4)	35(66)	1.65(0.87-3.03)	0.072		
Absent	8(18.6)	18(24)	0.72(0.51-1.02)			
**Glycated Haemoglobin**						
≥7%	40(93.0)	34(64.2)	1.88(1.40-2.53)	<0.001	6.21(1.61-24.01)	0.008
<7%(ref)	3(7.0)	19(35.8)	0.13(0.04-0.49)		1	
**Generalized Obesity**						
Present	20(46.5)	13(24.5)	1.61(1.02-2.56)	0.021	2.78(1.04-7.45)	0.012
Absent(ref)	23(53.5)	40(75.5)	0.37(0.16-0.89)		1	
**Central Obesity**						
Present	23(53.5)	25(47.2)	1.12(0.78-1.61)	0.341		
Absent	20(46.5)	28(52.8)	0.78(0.35-1.74)			
**Albuminuria**						
Present	36(85.7)	30(56.6)	1.75(1.26-2.41)	0.002	4.77(1.59-7.45)	0.005
Absent(ref)	6(14.3)	23(43.4)	0.22(0.08-0.60)		1	
**Dyslipidaemia**						
Present	43(100)	51(96.2)	1.84(1.53-2.22)	0.500		
Absent	0(0.0)	2(3.8)				

## Discussion

This study assessed and compared cardiovascular risk factors in elderly with T2DM and those without DM. It also determined cardiovascular risk factors associated with renal impairment in elderly with T2DM. All the cardiovascular risk factors assessed were significantly more prevalent in the elderly with T2DM. Obesity, albuminuria and glycated haemoglobin were significantly associated with renal impairment among the elderly with T2DM. The prevalence of renal impairment in the elderly with T2DM was 44.8%. The prevalence falls within the range of 38-62% reported among elderly in the Berlin Initiative Study [28]. However, it is higher than 35.7% reported among elderly in Finland [25]. The prevalence is however, lower than 50.2% reported by Drion *et al*. [29] among elderly with DM in Netherlands. The prevalence rates of central and generalized obesity in elderly T2DM group were 50% and 34.1%, respectively. The prevalence of *generalized obesity in our study is similar to 35%* reported in Spain by Gutiérrez-Fisac *et al*. [30]. It is higher than 17.9% reported in the ObEpi survey [31]. The prevalence of central obesity is similar to 47.6% reported in ObiEpi survey [31]. However, Gutiérrez-Fisac *et al*. [30] reported a higher value of 61.6%. The influence of lifestyle, environmental factor and genetics may be partly responsible for the different prevalence rates [32,33]. Obesity is associated with pro-inflammatory and oxidative stress state due to release of inflammatory substances such as interleukin-6, tumour necrosis factor-α and reactive oxygen species [34]. These substances lead to insulin resistance, hyperglycemia and atherosclerosis which account for increased cardiovascular morbidity and mortality [34].

Obesity was found to be a significant factor associated with renal impairment in elderly with T2DM in our study. This is similar to previous report by Mohammedi *et al*. [35] Obesity is associated with adverse renal outcomes such as albuminuria, reduction in GFR and progression to end stage renal disease among those with DM [35]. Obesity may cause glomerular hyperfiltration and glomerulosclerosis leading to progressive nephron loss, albuminuria, and reduction in the GFR [36,37]. The prevalence of dyslipidemia among elderly with DM was 97.9%. This is higher than 70.5% reported in Eritrea among an elderly population [38]. This is also higher than 89.1% reported by Ogbera *et al*. [39] in a population of patients with DM who were younger than our study participants. The effect of ageing on dyslipidemia may partly account for this difference [40]. Dyslipidemia is an established traditional cardiovascular risk factor for stroke, ischaemic heart disease and renal insufficiency in individuals with DM [10,41].

The prevalence of albuminuria among elderly with T2DM was 69.8%. This is higher than 25.7% and 47.5% reported in Iran and Netherlands, respectively [29,42]. This difference may be partly accounted for by the difference in age of the study participants and their duration of DM. The Iranian study participants were younger and had a shorter duration of DM than our study participants. In addition, differences in glycaemic control, genetic differences, presence of other cardiovascular factors such as hypertension, obesity, dyslipidemia may also affect the prevalence of albuminuria in individuals with DM [16,42,43]. Albuminuria significantly increases the risk of developing renal impairment in elderly patients with T2DM in our study. Albuminuria is a cardiovascular risk factor that is associated with progression of renal disease independent of blood pressure in individuals with T2DM [44].

Hypertension was present in 72.9% of elderly with T2DM. This is higher than the pooled prevalence of hypertension in Africa which was reported as 57% [7]. This finding supports the fact that DM predisposes to hypertension. DM increases peripheral vascular resistance and circulating volume which may lead to increase in blood pressure [45]. The prevalence of hypertension was lower than 86% reported in Cameroon where a lower cut-off of ≥ 130/80mmHg was used to define hypertension unlike our study that used a cut off of ≥ 140/90mmHg [46]. Hypertension was also more common among in elderly with T2DM with renal impairment compared to those with normal renal function. Hypertension is associated with reduction in GFR and albuminuria in T2DM [10]. Hypertension and DM cause atherosclerosis, vascular inflammation, endothelial dysfunction and structural remodeling [47]. The resulting vascular injury leads to various forms of cardiovascular diseases [47]. The prevalence of anaemia among elderly with T2DM was 53.1%. The prevalence is higher than 45.2 % reported by Awofisoye *et al*. [48] whose study participants were majorly middle aged. The age difference may partly account for this observed difference [48]. Anaemia is a non-traditional cardiovascular risk factor associated with increased risk of coronary event in the general population [49]. The interaction between iron deficiency, cytokine production and reduced renal perfusion are possible mechanisms responsible for the development of cardiovascular disease in anaemic individuals [50]. High glycated haemoglobin was present in 77.1% of the elderly with DM. Glycated haemoglobin is used conventionally to assess glycaemic control over a period of three months in individuals with DM. However, it also predicts cardiovascular risk in the general population [51]. Poor glycaemic control increases the risk of developing renal impairment in elderly with T2DM in our study. This is similar to a previous report by Russo *et al*. [10].

The limitation of the study is that cardiovascular diseases were not assessed in the study participants. To the best of our knowledge, this is the first study that provides information on cardiovascular risk factors and their association with renal impairment among elderly with DM in Nigeria. This information will provide foundation for more research in this evolving aspect of medicine in Nigeria and most parts of Africa.

## Conclusion

Cardiovascular risk factors were highly prevalent among elderly patients with T2DM compared with elderly without DM. Obesity, albuminuria and glycated hemoglobin were associated with renal impairment in elderly with T2DM. Early cardiovascular risk assessment and modification may reduce the cardiovascular and renal morbidity and mortality in elderly with T2DM.

### What is known about this topic

- The population of elderly with DM is on the increase globally;
- Cardiovascular disease is the leading cause of death among the elderly population.

### What this study adds

- Cardiovascular risk factors are highly prevalent in Nigerian elderly with T2DM;
- Some cardiovascular risk factors such as albuminuria, glycated hemoglobin and obesity were significantly associated of renal impairment in elderly with T2DM.
